# Comparison between Airway Responses to High versus Low Molecular Weight Compounds in Occupational Asthma

**DOI:** 10.1155/2011/781470

**Published:** 2011-05-25

**Authors:** D. Talini, F. Novelli, E. Bacci, F. L. Dente, M. De Santis, A. Di Franco, L. Melosini, B. Vagaggini, P. L. Paggiaro

**Affiliations:** ^1^Occupational Health Unit, Prevention Department, Galleria Gerace 14, 56126 Pisa, Italy; ^2^Cardio-Thoracic and Vascular Department, University of Pisa, 56126 Pisa, Italy

## Abstract

Occupational asthma (OA) is a heterogeneous disease, and the characteristics of the sensitizer responsible for OA may induce different clinical, functional, and biological manifestations. We examined the characteristics of 74 patients with OA induced by low molecular weight compounds (LMWC) or by high molecular weight compounds (HMWC) and diagnosed by specific inhalation challenge (SIC). Patients with OA induced by LMWC had a longer occupational exposure before the beginning of symptoms, a lower sputum eosinophilia, and a higher prevalence of late airway response (LAR), in comparison with patients with OA induced by HMWC. Pulmonary function tended to be poorer and atopy tended to be less frequent in LMWC-induced OA than in HMWC-induced OA. These data confirm and extend previous observations showing that the characteristics of the specific sensitizer inducing OA may determine different clinical, functional, and biological features, probably related to the difference pathogenetic mechanisms underlying these different types of OA.

## 1. Introduction

Occupational asthma with latency period can be induced by sensitization to either a specific allergen (high molecular weight compounds, HMWC) or chemical compounds (low molecular weight compounds, LMWC) present in the workplace [1]. The gold standard for the diagnosis of OA is represented by the Specific Inhalation Challenge (SIC) which is intended to demonstrate a direct relationship between exposure to a specific agent present in the workplace and an asthmatic response [2]. Few studies have analysed the variable patterns of response to HMWC and LMWC (early, dual, or late response) in limited numbers of patients, but how worker's characteristics may influence the pattern of response to the sensitising agents remains to be explored. Recent studies [3] have shown that there are significant differences in the type of airway changes induced by low and high molecular weight agents.

Sputum eosinophilia has been reported in a variable percentage of patients affected by occupational asthma, and some studies suggested that patients with asthma induced by LMWC may have a lower sputum eosinophil percentage than patients with asthma due to HMWC [4, 5]. Sputum eosinophils increase further after exposure to both HMWC and LMWC, showing the increase in allergic airway inflammation induced by these specific sensitizers [6]. Factors that influence the type of inflammatory responses are unclear but may include also the type of asthmatic reaction and the intensity of airway inflammation. In particular, it is not known if the type and/or the severity of airway inflammation may contribute to the determination of the pattern of airway response to the specific sensitizer.

We compared the clinical characteristics, the airway inflammatory pattern, and the model of specific airway response in patients with OA induced by HMWC or LMWC. The aim was to assess, in this specific model of asthma, whether the characteristics of the sensitizer and the different pathophysiologic mechanisms may be associated with a different asthma phenotype.

## 2. Materials and Methods

We studied 74 subjects with occupational asthma due to different sensitizers (diisocyanates, latex, hairdresser's products, wood, and flour dusts) observed consecutively in our asthma clinic: 48 were exposed to LMWC (isocyanates, persulfate salts, aziridine, and phenolic resins) agents, and 26 were exposed to HMWC (flour dusts, wood dusts, latex, detergents, and tobacco dusts). We selected only subjects in whom the diagnosis of occupational asthma had been performed by means of positive response to specific inhalation challenge (SIC). According to the international recommendations [2, 7], patients were all exposed to a known occupational sensitizer (Table 1) showed asthma deterioration at work and nonspecific bronchial hyperresponsiveness during a working period. 

Bronchial hyperresponsiveness was determined by methacholine challenge test performed as previously reported [8]; a provocative dose of a 20% decrease in FEV1 from baseline (PD20FEV1) of less than 1000 mcg was considered as positive for bronchial hyperresponsiveness. 

SIC was performed using different methods (Table 1): (a) for diisocyanates, subjects were exposed to vapours generated by blowing air on the surface of a small amount of toluenediisocyanate (TDI) or warming a small amount of methylenediphenyl diisocyanate (MDI) at 40°C, in a challenge chamber and monitoring isocyanate concentrations with a specific TDI/MDI detector (MDA model 7005 isocyanate detection equipment, MDA Scientific Inc., Glenview, IL); diluent was used as control exposure; the duration of the exposure was 30 min in a first test and 120 min in a second test (if the first resulted negative) [9]; (b) for dusts (flour, wood, persulfate, latex, and tobacco), subjects inhaled dusts by a mouthpiece connected to a small box where a suspension of the dust was obtained by blowing compressed air at 5 L/min through a bottle containing the dust; *lactose* powder was used as control test; the concentration of the dusts was measured by blowing air from the box through a cellulose nitrate filter of 0.8 *μ*m porosity by means of a vacuum pump [10]; (c) in two cases (one exposed to phenolic resins and the other to detergents), a realistic way was employed in order to simulate in laboratory the exposure of the workplace (spreading the substance on a small surface); diluent was used as control test, and the duration of exposure was still 30 minutes. In all SIC, FEV1 was measured immediately before and 5, 15, 30, and 60 minutes after the exposure to the sensitizer, then hourly for 8 hours. A positive response was defined as a decrease in FEV1 from baseline of more than 15% during the first hour (immediate response) or between the second and the 8th hour (late response), and in absence of a more than 10% decrease in FEV1 during a control test performed in a different day with diluent (for diisocyanates or other simple chemicals) or with lactose dust (for other dust sensitizers).

One or two weeks before challenge, other measurements at diagnosis included skin prick tests to common allergens (to check for atopy), and collection of sputum induced by the inhalation of saline solution. The method for induction and processing has been previously described [11]. Total and differential counts of inflammatory cells (eosinophils, macrophages, neutrophils, and lymphocytes) were considered; we chose 2% as the upper limit of normal range for sputum eosinophils [12]. 

All patients gave their informed consent to the management of their personal data.

Characteristics of subjects (age, sex, smoking habit, atopy, duration of symptoms and exposure, latency, type of response, sputum eosinophilia, and functional data) were compared between two groups with asthma induced by HMWC or LMWC.

Descriptive analysis for data collected at diagnosis was performed, with data expressed as mean (+standard deviation, SD) or median (range) for normally and nonnormally distributed data, respectively. PD20FEV1 methacholine was reported as geometric mean and log-transformed for statistical analysis. Comparison among groups was performed by appropriate parametric (analysis of variance, Chi-square test, and unpaired *t*-test) and nonparametric tests (Mann-Whitney *U* test). A *P* value lower than 5% was considered as significant, and a *P* value between 0.1 and .05 was considered as expression of a trend.

## 3. Results

Patients' characteristics are summarized in Table 2. These characteristics were similar in both groups with asthma induced by HMWC and LMWC, except for duration of exposure, latency period, and sputum eosinophilia. Duration of exposure and latency periods were higher in subjects with asthma due to LMWC, who had also a lower sputum eosinophilia. FEV1 was lower in absolute value, but not in percentage of predicted, in patients with LMWC- than in patients with HMWC-induced asthma. Atopy was more frequently observed in patients with HMWC- than in patients with LMWC-induced asthma, but the difference was not statistically significant. Atopic subjects had a higher FEV1 (*P* = .02) and a higher percentage of eosinophils (*P* = .005).

The comparison among groups of subjects with asthma due to different sensitizers was strongly affected by the low number of subjects included in the different groups, except for patients sensitized to diisocyanates (*N* = 37) and to flour dust (*N* = 20) who were different for age, atopy, duration of exposure, and sputum eosinophil percentage, in the same way as the difference between LMWC and HMWC. There was also a difference in the gender, related to the specific jobs (e.g., female in the subjects exposed to persulfate, or male in subjects exposed to diisocyanates).

**Figure 1:**
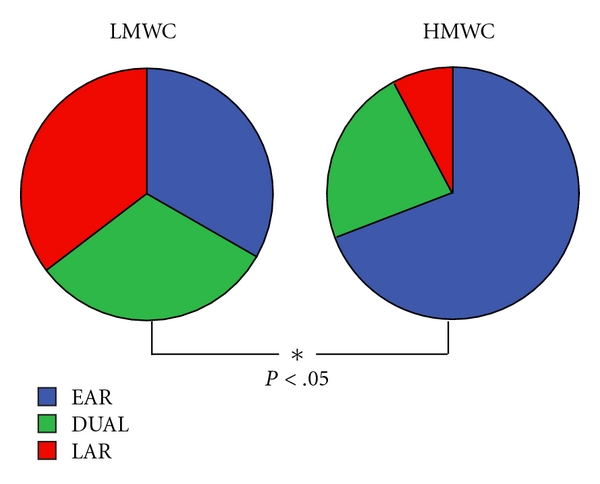
Type of response to SIC in subjects sensitized to high and low molecular weight agents. (EAR: early response, LAR: late response).

**Figure 2:**
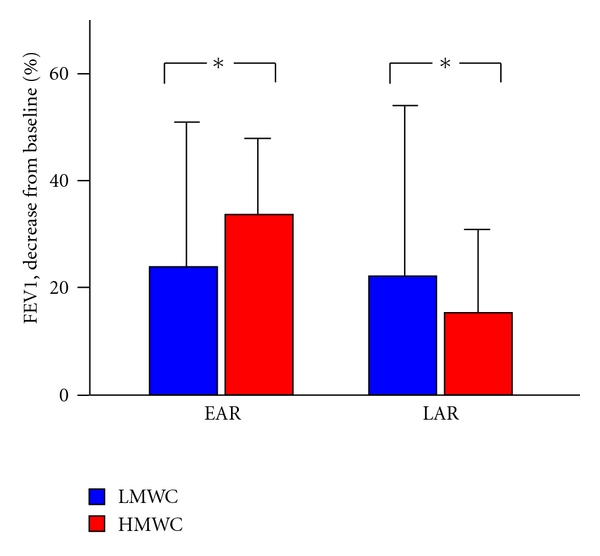
Magnitude of the early (EAR) and late (LAR) responses (expressed as percent decrease in FEV1 from baseline) during SIC to low (LMWC) or high (HMWC) molecular weight compounds, *P < .05.

Patterns of response following SIC were different for HMWC and LMWC. Subdividing subjects by type of response, immediate responses (early + dual response) were common in subjects exposed to HMWC (Figure 1). Also, in subjects with higher sputum eosinophilia, immediate responses were higher (61.1% versus 94.1%, *P* = .02). Considering all types of responses, magnitude of the early (EAR) responses was higher during SIC to LMWC while the magnitude of the late (LAR) responses was higher during SIC to HMWC (Figure 2).

## 4. Discussion

The present study shows that few clinical characteristics may differentiate patients with occupational asthma induced by LMWC from those with asthma induced by HMWC. In particular, duration of exposure before the beginning of asthma symptoms and the severity of the eosinophilic airway inflammation were the only findings which differentiated two groups of patients. In the same way, the pattern or airway response was consistently different, with patients with LMWC-induced asthma showing a higher frequency and a greater severity of the isolated LAR than patients with HMWC-induced asthma who showed, on the contrary, a greater frequency and severity of the early response. These data confirm and extend previous observations confirming that some clinical and functional characteristics are different between subjects with occupational asthma induced by LMWC or by HMWC.

Our observations concern a wide range of sensitizing agents and a consistent number of subjects, as in other previous few studies [3, 13]. Differently from other previous papers which have considered only a subset of baseline measurements, our data include several clinical and functional findings which may be measured in these patients, including also the evaluation of the level of eosinophilic airway inflammation and the pattern of response to SIC. In effect, previous studies have shown that patients with LMWC-induced asthma had a greater duration of exposure to the specific sensitizer before the beginning of asthma symptoms than patients with HMCW-induced asthma [14, 15], or that LMWC asthma showed less frequent sputum eosinophilia [5] or a lower frequency of immediate airway response to SIC [3, 16]. All these data have been confirmed in our study which included a large set of clinical, functional, and biological findings of the disease at the baseline assessment.

However, other studies did not show relevant difference in terms of type and level of airway inflammation, level of asthma severity at the diagnosis, or rate of recovery from asthma after work cessation, between asthma induced by HMWC or LMWC [17–20]. However, some of these studies included small number of subjects, with several confounding factors (like the persistence or removal from exposure, or the severity of the disease).

This difference in clinical, functional, and biological features is probably related to the different pathophysiologic mechanisms underlying HMWC and LMWC occupational asthma [21]. Despite the large heterogeneity of the pathogenetic mechanisms underlying this disease, OA induced by HMWC is sustained by an IgE-mediated mechanism, which is well know to induce immediate airway reaction, due to mastcell activation which initiates the inflammatory cascade leading to a late reaction and the recruitment of eosinophils in the airways. Differently, LMWC elicit a lymphocyte-specific sensitization, with predominant isolated late response and lower eosinophilic inflammation. These features, in addition to the longer duration of exposure before the beginning of symptoms and the lower pulmonary function, make LMWC-induced asthma as a good model of late-onset asthma, different from HMWC-induced asthma which has many findings more typical of early-onset asthma. The hypothesis of different characteristics and outcome between early- and late-onset asthma has been suggested by other authors [22] and might be further supported by the different outcome of asthma after removal from work exposure. In effect, independently from the specific characteristics of the occupational agent, patients who at diagnosis had higher levels of sputum eosinophila reported a better outcome in the followup than patients without sputum eosinophilia, probably because a better response to the corticosteroid treatment [23].

## 5. Conclusions

In summary, we may speculate that the chemical characteristics of the specific sensitizer responsible for OA are responsible for a different pathophysiologic mechanism, which may determine different clinical, functional, and biological manifestation of the disease, including the pattern of specific airway response. In this way, occupational asthma may represent a good model for studying the heterogeneity of asthma and the difference between early- and late-onset asthma.

## Figures and Tables

**Table 1:** Characteristics of the compounds used for performing specific inhalation tests (SIC), concentrations used during SIC and duration of the exposure.

Agent	Control	Number of subjects	Challenge concentration	Time exposure
TDI vapours	Diluent	37	0.002–0.003 ppm	30–120′
Flour dust	Lactose dust	20	0.3–0.5 mg/m^3^	30′
Wood dusts	Lactose dust	3	0.3–0.5 mg/m^3^	30′
Persulfate salts	Lactose dust	6	0.05–0.1 mg/m^3^	30′
Aziridine	Lactose dust	2	0.03–0.05 mg/m^3^	30′
Latex solution	Normal saline	3	<0.0001 mg/m^3^	30′
Tobacco dusts	Lactose dust	1	0.3–0.5 mg/m^3^	30′
Phenolic resins and Detergents vapours	Diluent	2	Not measured*	30′

*Subjects simulated the job activity in laboratory.

**Table 2:** Characteristics of the patients examined, according to the type of the occupational sensitizer.

	LMWC	HMWC
Patients	48	26
Age, years	43.8 ± 12.0	38.9 ± 10.8
Gender,		
Male	34 (70.8)	20 (76.9)
Female	14 (29.2)	6 (23.0)
Atopy	12 (25)	12 (46.1)
Smoking habit		
Nonsmokers, *N* (%)	23 (47.9)	14 (53.8)
Smokers, *N* (%)	4 (8.3)	2 (7.7)
Ex-smokers, *N* (%)	21 (43.8)	10 (38.4)
Therapy *N*/*Y*	29/19	14/12
Duration of symptoms, yrs	6.1 ± 6.9	6.4 ± 5.7
Duration of exposure, yrs	20.1 ± 13.1	15.2 ± 7.7*
Latency period, yrs	13.9 ± 12.7	8.7 ± 5.7*
Baseline FEV1		
L	3.01 ± 0.67	3.39 ± 0.68*
% pred	89.2 ± 16.0	93.5 ± 14.8
Baseline PD20FEV1 (mg)	0.22 (0.01–5.00)	0.18 (0.02–5.00)
Sputum eosinophilia		
Eosinophils, %	0.95 (0–32)	6.8 (0–43.1)*

Data are presented as *n* (%), M ± SD or GM (range; PD20FEV1) or median (range; eosinophils, %).

*P < .05.

LMWC: low molecular weight compound; HMWC: high molecular weight compound.
